# Highly pathogenic bovine viral diarrhea virus BJ-11 unveils genetic evolution related to virulence in calves

**DOI:** 10.3389/fmicb.2024.1540358

**Published:** 2025-01-14

**Authors:** Yuanyuan Zhang, Jing Cheng, Yu Guo, Yibin Hu, Zhuo Zhao, Wenxiao Liu, Linyi Zhou, Peize Wu, Chunjie Cheng, Chun Yang, Jing Yang, Enqi Du, Yongqing Li

**Affiliations:** ^1^Institute of Animal Husbandry and Veterinary Medicine, Beijing Academy of Agriculture and Forestry Sciences, Beijing, China; ^2^College of Veterinary Medicine, Northwest A&F University, Yangling, China; ^3^The College of Veterinary Medicine, Hebei Agricultural University, Baoding, China; ^4^Beijing Centrebio Biological Co., Ltd., Beijing, China; ^5^College of Animal Science and Technology, Jiangxi Agricultural University, Nanchang, China; ^6^Animal Science and Technology College, Beijing University of Agriculture, Beijing, China

**Keywords:** bovine viral diarrhea virus, lethal strain, BVDV 1b, glycoprotein mutations, evolve, vaccination strategies

## Abstract

Bovine viral diarrhea virus (BVDV) is the causative agent of bovine viral diarrhea, which causes significant economic loss to the global livestock industry. Despite the widespread use of inactivated BVDV vaccines, highly pathogenic strains continue to emerge. In China, regional variations in BVDV subtypes, morbidities, and symptoms, however, only the BVDV 1a subtype vaccine is currently approved. Therefore, this study is to gain insight into the biological characteristics and genetic variation of BVDV strains prevalent in Beijing. Meanwhile, this will provide a theoretical foundation and technical support for the prevention and control of BVDV, as well as raise awareness of the potential for virulence enhancement caused by the unregulated use of BVDV vaccines. In this study, A BVDV strain, BJ-11, was isolated from calves that died of diarrhea and vaccinated of BVDV. To evaluate its virulence, newborn calves were experimentally infected with the BJ-11. Clinical signs included fever, diarrhea, bloody stools, anorexia, and death in some cases. A marked reduction in leukocyte and lymphocyte counts were observed, accompanied by an increase in neutrophil counts. Histopathological changes manifested as severe lung lesions. Phylogenetic analysis indicated that BJ-11 belongs to the BVDV 1b subtype, genetically closest to the JL-1 strain. Analysis of the E2 glycosylation site disappeared (298SYT) in one of the four common glycosylation sites in the BVDV-1, which has been reported to affect the ability of the virus to infect and an additional glycosylation site (122NGS). These results indicate that BJ-11 is a highly pathogenic strain evolved from a low-virulence ancestor and should be served as a challenge strain. Simultaneously, these results contribute to a broader understanding of BVDV and whether imperfect vaccination strategies lead to reversal of immunosuppressive virulence.

## 1 Introduction

Bovine viral diarrhea (BVD) is an acute infectious disease caused by the bovine viral diarrhea virus (BVDV) (Liu et al., 2021). Cattle of all breeds and ages serve as natural hosts of BVDV, facilitating its transmission to other species such as sheep, pigs, and various wildlife (de Oliveira et al., 2020; Mishra et al., 2009; Nelson et al., 2015). Clinical signs of BVD include fever, diarrhea, leukopenia, poor reproductive performance and immune dysfunction. Furthermore, the BVDV infection leads to persistent infection (PI) and immunosuppression (Lunardi et al., 2008; Oguejiofor et al., 2019; Abdelsalam et al., 2023). Immunosuppression, in turn, results in a decline in key immune components, such as white blood cells (WBC), lymphocytes (Lym), neutrophils (Neu) and platelets, leaving the animal vulnerable to other diseases (Larson, 2015). In the context of BVDV transmission, PI cattle are the primary reservoir for BVDV transmission within herds and to other species (Khodakaram-Tafti and Farjanikish, 2017). Vaccination remains a critical strategy for controlling BVD. In China, only the inactivated BVDV vaccine is currently licensed for use, which offer significant safety advantages but fail to provide complete homologous and cross-protection. As a result, BVDV continues to impose a substantial economic burden on the global livestock industry.

As a single-stranded and positive-sense RNA virus, BVDV, together with classical swine fever virus (CSFV) and Border disease virus (BDV), belong to the genus *Pestivirus* of the family *Flaviviridae* (Saltik et al., 2022). The BVDV genome, approximately 12.3 kilobases in size, encodes four structural proteins (capsid, Erns, E1, and E2) and eight non-structural proteins (Npro, p7, NS2, NS3, NS4A, NS4B, NS5A, and NS5B) (Chang et al., 2021). Structural proteins can bind to both the genomic RNA and lipid bilayers, thereby facilitating viral particle formation. Non-structural proteins, on the other hand, play critical roles processes including viral replication, transcription, and translation, either independently or in coordination with other proteins (Chi et al., 2022). The BVDV genome also comprises untranslated regions (UTR) at both its 5′ and 3′ ends. The 5′UTR in involved in RNA replication and functions as the internal ribosome entry site (IRES), while the 3′UTR contains structural elements that are essential for RNA replication and the highly conserved binding sites for microRNAs, miR-17 and let-7. Phylogenetic analyses offer insights into the evolutionary history and diversity of BVDV (Cornwell and Nakagawa, 2017). The 5′UTR is highly conserved, which is commonly used for phylogenetic analyses and genotyping of BVDV. However, its limited length and variability, the use of other genome regions, such as Npro, E2, for more comprehensive evolutionary analyses (Gomez-Romero et al., 2021; Tautz et al., 2015; Abe et al., 2016; Zhu et al., 2022).

BVDV is classified into two biotypes, non-cytopathogenic (NCP) and cytopathogenic (CP), and three genotypes: BVDV-1, BVDV-2, and BVDV-3 (Shah et al., 2022). To date, at least 23 different sub-genotypes of BVDV-1 (BVDV-1a–BVDV-1v) and four sub-genotypes of BVDV-2 (2a–2d) have been identified (Yeşilbağ et al., 2014; Deng et al., 2020). Among these, BVDV-2a is the most widely distributed worldwide, whereas 2b, 2c, and 2d are largely confined to South America. In China, the BVDV-1 subtypes, such as 1a, 1b, 1c, 1d, 1 m, 1o, 1q, and 1u, are the predominant in cattle herds, while BVDV-2 is more prevalent in western of China (Deng et al., 2020; Deng et al., 2015; Yeşilbağ et al., 2017; Zhong et al., 2011). The emergence of new viral subtypes is driven by international cattle trade, which facilitates viral recombination and mutation. Vaccination remains the most widely used strategy against BVDV because of its relatively low cost and ease of implementation. However, current vaccines are limited their ability to prevent the establishment of PIs in cattle. Additionally, immune pressure increases virulence and genetic diversity of BVDV. Highly pathogenic strains of BVDV have emerged more frequently in Chinese cattle herds in recent years, likely due to extensive use of immunization with inactivated BVD vaccines (Zhang et al., 2022; Wang et al., 2020). Therefore, these challenges highlight the need for further investigation into BVDV infections, particularly with respect to their genetic evolution, virulence, and the development of more effective preventive measures (Su et al., 2022).

Currently, the only commercially available BVDV vaccine in China is based on the BVDV-1a subtype. However, the distribution of prevalent subtypes, associated morbidities, and clinical symptoms vary significantly across different regions. This study aimed to isolate and evaluate the virulence of BVDV strains from Beijing, focusing on understanding the pathogenicity and genetic characteristics of currently prevalent strains. Additionally, this research sought to analyze the underlying factors contributing to the increased virulence of this strain.

## 2 Materials and methods

### 2.1 Virus detection and isolation

Field samples were collected from the feces of calves suffering from diarrhea and death. The RNA was extracted using an E.Z.N.A.® Viral RNA Kit (Omega Bio-tek, Norcross, GA, USA), following the manufacturer’s instructions. cDNA was synthesized using an RNA reverse transcription kit and tested by reverse transcription-polymerase chain reaction (RT-PCR). DNA was extracted using the E.Z.N.A.® Viral DNA Kit (Omega Bio-tek). A series of primers was designed for the detection of pathogens, including BVDV, bovine herpesvirus 1 (BoHV-1), bovine rotavirus A/C (BRoV A/C), bovine rotavirus B (BRoV B), bovine coronavirus (BCoV), bovine parainfluenza virus 3 (BPIV-3), bovine respiratory syncytial virus (BRSV), and *Mycoplasma bovis* (*M. bovis*) (Supplementary Table S1) (Thanthrige-Don et al., 2018). The fecal samples were diluted with PBS to be 10% (w/v) suspensions. The suspension was vortexed thoroughly and centrifuged at 4500 g for 10 min at 4°C. The supernatant was filtered through 0.22 mum syringe filter (Millipore, Billerica, MA) and stored at −80°C as an inoculum for virus isolation until use. Virus isolation of BVDV was attempted on Madin–Darby bovine kidney (MDBK) cells which cultured in Dulbecco’s modified Eagle’s medium (DMEM) (Gibco, Grand Island, NY, USA), supplemented with 10% (v/v) heat-inactivated fetal bovine serum (FBS) (Gibco), at 37°C with 5% CO_2_. After incubation at 37°C the supernatant from BVDV-positive sample was filtered and inoculated the MDBK cells, then incubated for 1 h and 5% CO_2_ and then the DMEM containing 2% FBS was replaced. After 72 h, the infected cells were frozen and thawed three times, and the supernatant was collected for the next infection.

### 2.2 Indirect immunofluorescence assay (IFA)

MDBK cells were infected with BJ-11 or were mock-infected. After 72 h, the cells were fixed with 4% paraformaldehyde, permeabilized with 0.1% Triton X-100, and blocked with 5% bovine serum albumin (BSA). Subsequently, the cells were incubated with BVDV monoclonal antibodies, goat anti-mouse IgG/FITC (Invitrogen, Carlsbad, CA, USA), and 4′,6-diamidino-2-phenylindole. The stained cells were observed under an Olympus DMi8 fluorescence microscope (Olympus Corporation, Tokyo, Japan).

### 2.3 Viral growth kinetics of BVDV BJ-11

To evaluate the one-step growth dynamics of BVDV BJ-11, MDBK cells were infected with the virus. Samples were collected at 0, 4, 8, 12, 16, 20, 24, 32, 40, 48, 60, 72, and 96 h post-infection (hpi). Simultaneously, the virus was propagated through 10 consecutive passage to access its stability. The viral titers at each time point and across generations were measured using the median tissue culture infectious dose (TCID_50_), based on the Reed-Muench calculation. The TCID_50_ values were confirmed by using IFA.

### 2.4 Animals and experimental design

Six healthy newborn Holstein calves were randomly assigned to two groups (three calves per group). Blood samples were collected for the detection of BVDV antigens and antibodies. For this study, 6 mL of 10^6^ TCID₅₀/mL of BVDV BJ-11 virus or DMEM (control group) were administered nasally and intramuscularly. Rectal temperature and clinical signs were monitored daily. Clinical scores were assigned from 1 to 14 dpi including conjunctivitis, nasal discharge, coughing, abnormal breathing, diarrhea, and loss of appetite (Supplementary Table S2). Symptom severity was quantified using a scoring system ranging from 0 to 3, with higher scores indicating a greater degree of severity (Zhu et al., 2022). Serum, nasal swab (NSs), and rectal swab (RSs) samples were collected at 1, 3, 5, 7, 9, 11, and 13 dpi to determine rectal viral loads. Fresh blood samples were assayed for WBC, Lym, Lym%, and Neu counts. Viral loads in the NSs, RSs, and blood were determined by quantitative RT-qPCR using specific primers (Supplementary Table S1). Calves were euthanized at 21 dpi, and tissue samples were collected for histopathological and immunohistochemical examinations.

### 2.5 Detection of viral load via RT-qPCR

Determination of the systemic distribution of BVDV. Viral RNAs levels were determined by RT-qPCR in different tissues from dead or euthanized calves at 21 dpi. Viral RNAs was extracted from the organs of the calves (heart, lung, liver, kidney, spleen, duodenum, jejunum, liver, tonsils, superficial cervical lymph nodes, mesenteric lymph nodes, inguinal lymph nodes, rumen, and abomasum).

### 2.6 Histopathological examination

The tissues from lung, duodenum, spleen, inguinal lymph nodes, and tonsils were fixed in 10% neutral buffered formalin for 72 h after being embedded in paraffin and then sectioned into 4–5 μm-thick slices. These sections were stained with hematoxylin and eosin (H&E) to enable histopathological examination.

### 2.7 Viral genome sequence

The complete genomic sequence of the BVDV strain was assembled using the next-generation sequencing (NGS) method (Colitti et al., 2019). The BVDV genome sequence was submitted to GenBank (accession number PP213451.1).

### 2.8 Nucleotide and amino acid analyses

The complete genome sequences and deduced amino acid sequences were analyzed, and their homology was compared with ten representative BVDV strains using the ClustalW method in Lasergene (Version 7.1) (DNASTAR Inc., Madison, WI, USA).

### 2.9 Multiple alignments, phylogenetic, and recombination analyses

To evaluate the genetic similarity and evolutionary characteristics of BVDV BJ-11, complete genome and fragment alignments were conducted using DNAMAN 7.1 software.^1^ A total of 33 representative BVDV genomes were obtained from GenBank for comparison. Sequence alignment was carried out using ClustalW and neighbor-joining analysis were constructed with Molecular Evolutionary Genetics Analyses (MEGA) X (Kumar et al., 2018). The reliability of the phylogenetic tree was evaluated by bootstrapping with 1,000 replicates (Morrison, 1996). Potential recombination events in the BJ-11 isolate were screened using the genomes aligned with RDP4, which incorporates seven methods: RDP, GENECONV, BootScan, Maxchi, Chimaera, SiScan, and 3Seq (Martin et al., 2015). Similarity analyses were performed using the SimPlot software package (version 3.5.1)^2^ for potential recombinant events. Glycosylation site prediction was performed using an online website.^3^ The tertiary structure of E2 was predicted using I-TASSER,^4^ and surface representations of the tertiary structure were generated using PyMOL.^5^

### 2.10 Statistical analyses

All data were analyzed using GraphPad Prism 8.3.0 software (GraphPad, La Jolla, CA, USA) and presented as mean ± standard deviation (SD). Statistical significance was calculated using the student’s *t*-test for one comparison and analysis of variance (ANOVA) for multiple comparisons. Statistical significance was evaluated by determining the *p*-values using a two-tailed Student’s *t*-test.

## 3 Results

### 3.1 Detection, isolation, identification, titer, and one-step growth curve of BVDV isolate

**Figure 1:**
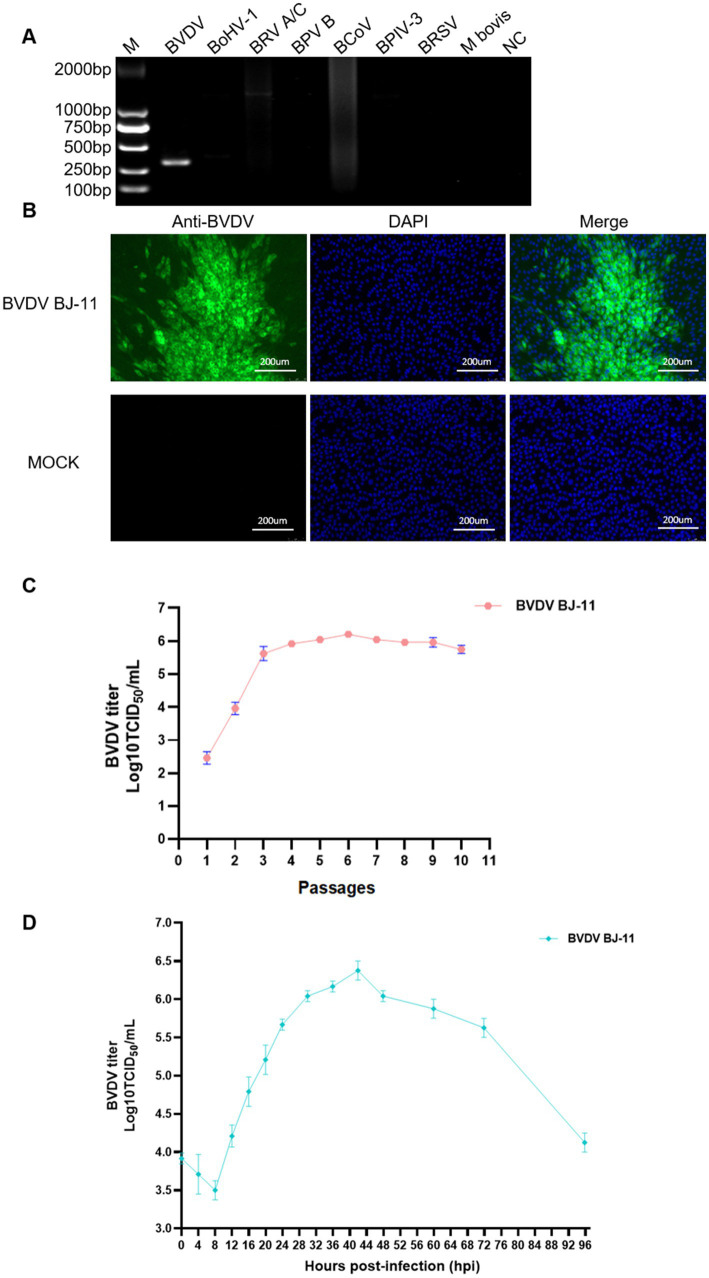
Identification of BVDV BJ-11 isloate. **(A)** PCR detection of BVDV. PCR amplification was employed to identify the causative agent, utilizing primers specific to BVDV, BoHV-1, BRoV A/C, BRoV B, BCoV, BPIV-3, BRSV, and *M. bovis*. **(B)** Immunofluorescence assay (IFA) of MDBK cells infected with BJ-11 at 72 hpi. MDBK-infected cell inoculated with BVDV mAb. **(C)** The median tissue culture infectious dose (TCID50) of the BJ-11 strain in passages. **(D)** One-step growth curves of BVDV BJ-11 strain.

The fecal sample was detected as a single positive by RT-PCR and PCR (Figure 1A). After three passages, no obvious CPEs were observed in the inoculated MDBK cells, but specific green signals observed through IFA (Figure 1B) which was defined as non-cytopathic (NCP) and named BJ-11. The titer of the BVDV isolate increased from 10^2.5^ TCID₅₀/mL to 10^6.125^ TCID₅₀/mL with the increase of passages and stabilized at 10^6^ TCID₅₀/mL (Figure 1C). One-step growth curves showed that viral replication reached its peak level at 42 hpi (Figure 1D).

### 3.2 Clinical signs

**Figure 2:**
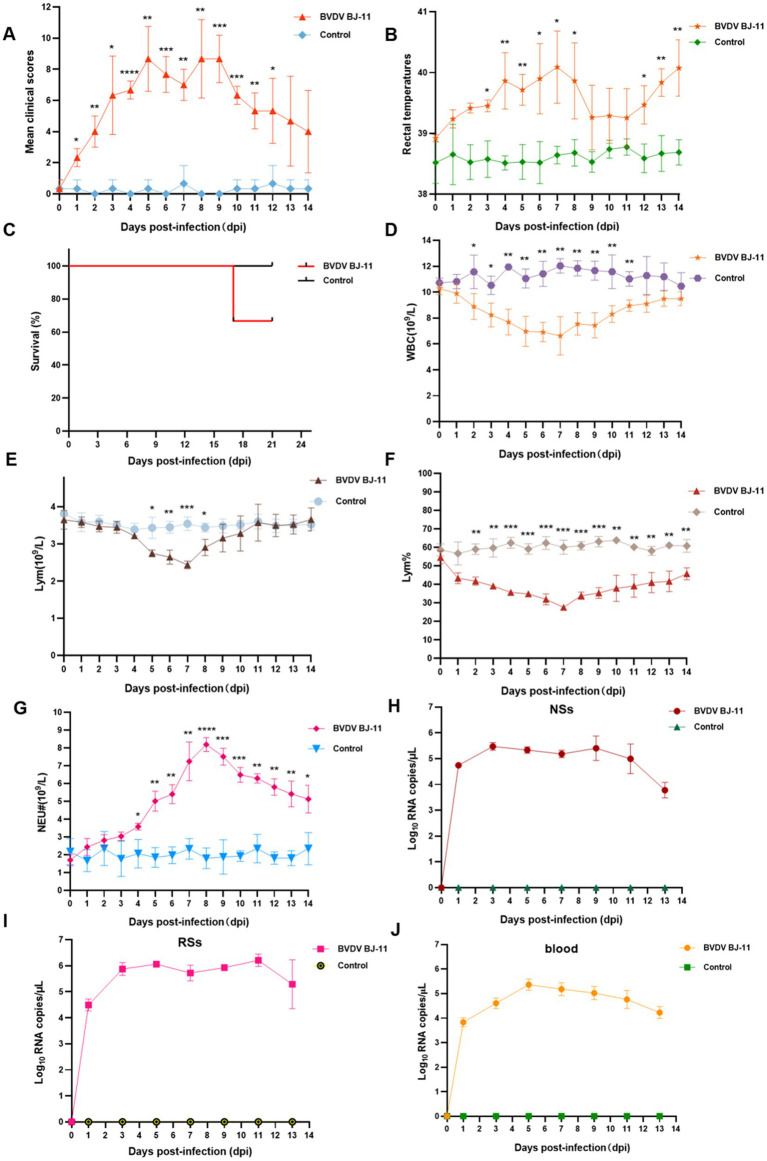
Pathogenic and serological characteristics of BVDV BJ-11 isolate. **(A)** Clinical signs. **(B)** Rectal temperature. **(C)** Survival rate. **(D–G)** Complete blood cell count. **(D)** White blood cell (WBC), **(E)** Lymphocytes (Lym), **(F)** Lymphocytes percent (Lym%) and **(G)** Neutrophils (Neu). **(H–J)** Relative quantification of virus load. **(H)** Blood, **(I)** nasal swabs (NSs), and **(J)** rectal swabs (RSs). Statistical significance was evaluated by determining *p*-values using the 2-tailed Student’s *t*-test. *p < 0.05, **p < 0.01, ***p < 0.005, ****p < 0.001.

The replication and pathogenesis of BVDV were determined in calves and all animals were subjected to daily monitoring. Observed symptoms including high fever, shortness of breath, sneezing, runny nose, anorexia, conjunctivitis, depression, and lethargy. The feces were initially watery and gray in color, with an unpleasant odor. Subsequently, the animals were observed to have discharged bloody stools mixed with mucus. Clinical symptom scores were higher at 5, 8, and 9 dpi, as illustrated in Figure 2A. Furthermore, a biphasic fever was observed, reaching 40.7°C at 7 dpi, followed by temperatures decline. However, the temperature began rising again at 12 dpi (Figure 2B). One calf in the experimental group died at 16 dpi (Figure 2C). In contrast, no clinical symptoms were observed in the control group throughout the study.

### 3.3 Hematological analyses

Blood samples were collected for analysis, revealing notable changes in WBC counts in the challenge group. WBC counts began to decline at 3 dpi, reaching lowest point (6.62 × 10^9^/L) at 11 dpi, with a statistically significant difference identified (*p* < 0.01). Subsequently, a rebound was observed, indicating a recovery in WBC counts. Statistically significant difference was observed between the challenge and control groups at 2 and 11 dpi (*p* < 0.05) (Figure 2D). Lym counts also showed a significant decline at 5 dpi, reaching their lowest levels at 7 dpi (*p* < 0.01) (Figure 2E). Subsequently, a rebound was observed. In contrast, a more pronounced difference in Lym% was evident, as illustrated in Figure 2F. Lym% dropped at 2 dpi and reached 27% compared 60.1% in the control group, at 7 dpi, exhibiting a statistically significant difference (*p* < 0.001) between 4 and 9 dpi. An increase in Neu counts occurred at 4 dpi (*p* < 0.05), peaking at 8.19 × 10^9^/L at 8 dpi. This was 4.55-fold increase compared to the control group (1.8 × 10^9^/L), with a remarkably significant difference (*p* < 0.0001). To evaluate viral shedding and viremia, NSs and RSs were collected every 2 days. The highest viral load in NSs was detected at 3 dpi, as illustrated in Figure 2H. In contrast, the viral load was consistently higher in the RSs than in the NSs, with the highest viral load observed at 11 dpi (Figure 2I). Viremia levels peaked at 5 dpi (Figure 2J).

### 3.4 Tissue tropism of viral RNA

**Figure 3:**
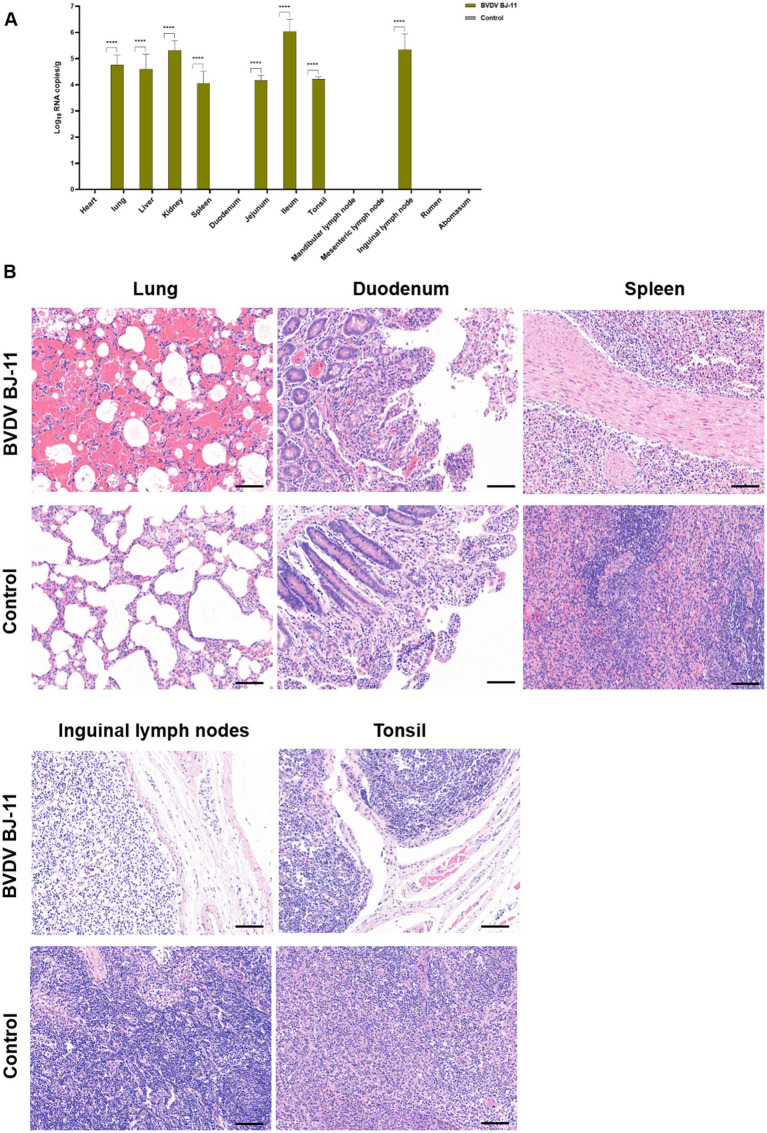
Viral load, and histological lesions. **(A)** Tissue samples including heart, lungs, liver, kidneys, spleen, duodenum, jejunum, ileum, tonsils, mandibular lymph nodes, mesenteric lymph nodes, inguinal lymph nodes, rumen, and stomach were collected to determine the copy number of BVDV by RT-qPCR. **(B)** Histopathological of lungs, duodenum, spleen, inguinal lymph nodes and tonsils. The horizontal line on the bottom right of each figure is the scale bar (100 μm scale). Statistical significance was evaluated by determining *p*-values using the two-tailed Student’s *t*-test. *p < 0.05, **p < 0.01, ***p < 0.005, ****p < 0.001.

To investigate the tissue tropism of virus, samples were collected from various organs and tissues, including heart, lung, liver, kidney, spleen, duodenum, jejunum, ileum, tonsils, mandibular lymph nodes, mesenteric lymph nodes, inguinal lymph nodes, rumen, and stomach. RT-qPCR analysis revealed the virus was mainly distributed in the spleen, jejunum, ileum, tonsils, and inguinal lymph nodes (Figure 3A).

### 3.5 Histopathological examination

Pathological examination revealed lesions primarily in the lungs, gastrointestinal tract, and lymphatic tissues. In the lungs, hemorrhages were evident accompanied by notable thickening of the alveolar septa. Additionally, there was considerable infiltration of inflammatory cells accompanied by a considerable quantity of exudate within the alveolar lumen and discernible destruction of the alveolar structure. The duodenum displayed apical shedding of villi, inflammatory cell infiltration of the lamina propria, and evidence of bruising. The spleen exhibited hemorrhagic changes accompanied by white marrow atrophy and structural disruption of the white and red medullae oblongata. The inguinal lymph nodes exhibited severe edema, a loose cortical structure, and lymphocytopenia, and the lymph node germinal centers were not visible. The tonsils demonstrated a reduction in lymphocytes and sparse tissue structure. No histopathological damage was observed in any tissues from the control animals (Figure 3B).

### 3.6 Genomic and phylogenetic analyses

**Figure 4:**
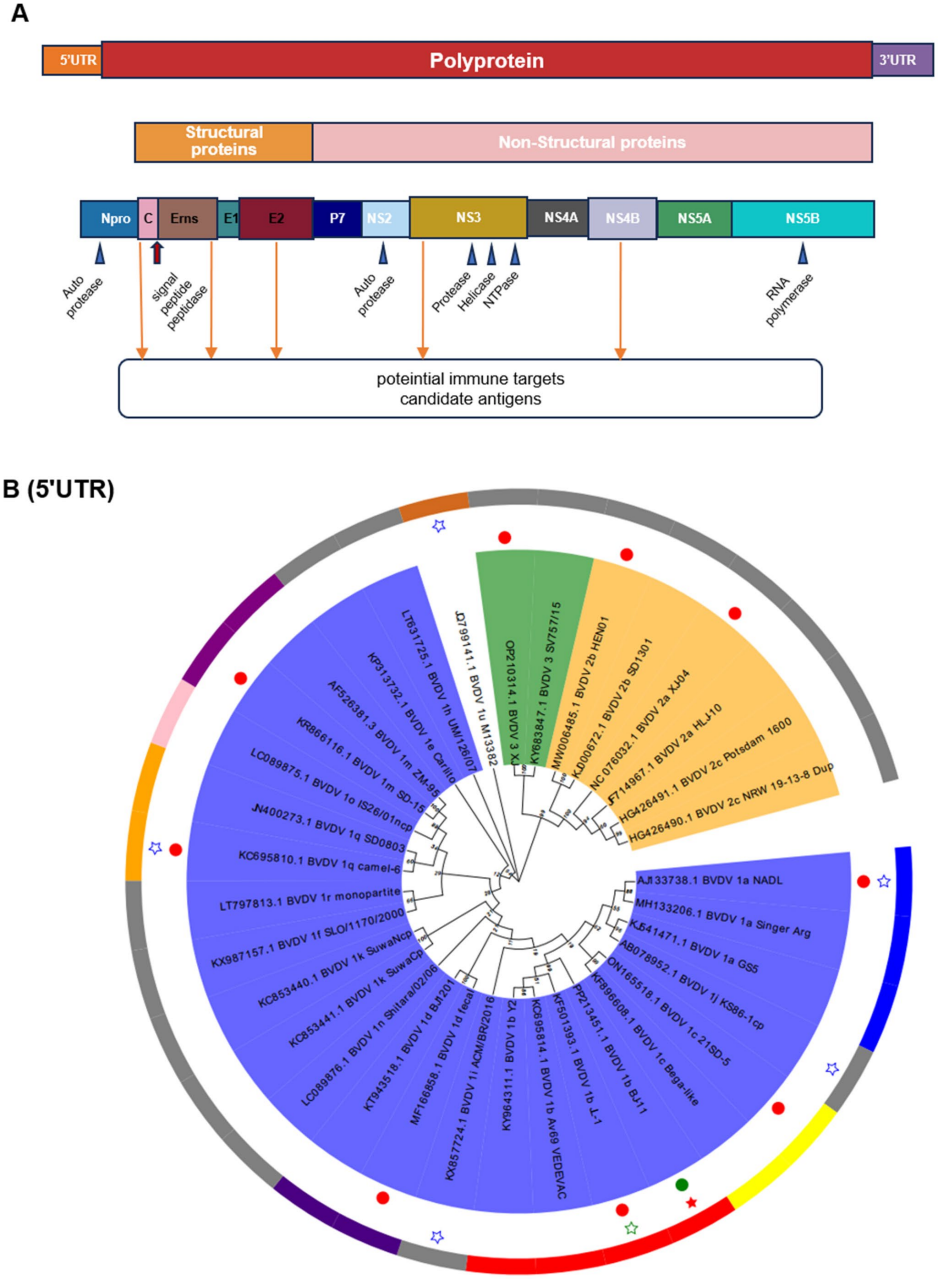
Schematic diagram of the genomic structure and phylogenetic analysis. **(A)** Schematic diagram of BVDV genome. **(B)** Phylogenetic analysis of BVDV isolates and reference strains based on the 5-untranslated region (5’UTR). The tree was constructed based on 5’UTR by the neighbor-joining method using the MEGA X and 1,000 bootstrap replicates. BJ-11 isolate is indicated by a red pentagram. The 10 BVDV strains used in homology and recombination analysis indicated in solid circles.

The complete genome contained 12,246 nucleotides (nt). The genome structure (Figure 4A), including 5′UTR of 382 nt, 3′UTR of 167 nt, and an open reading frame (ORF) encoding a large precursor polyprotein of 3,898 amino acids (aa) (Supplementary Table S3). To identify the genotypes, the BJ-11 and 33 representative strains were used to construct phylogenetic trees. Firstly, the sequence of 5′UTR from this study underwent alignment with corresponding sequences constructed from other BVDV strains (Figure 4B). Phylogenetic analysis identified BJ-11 as BVDV-1b. Concurrently, the evolutionary trees of the complete genome, Npro, NS3, E0, and E2 (Supplementary Figures S1A–E) yielded identical results.

### 3.7 Homology analysis

**Figure 5:**
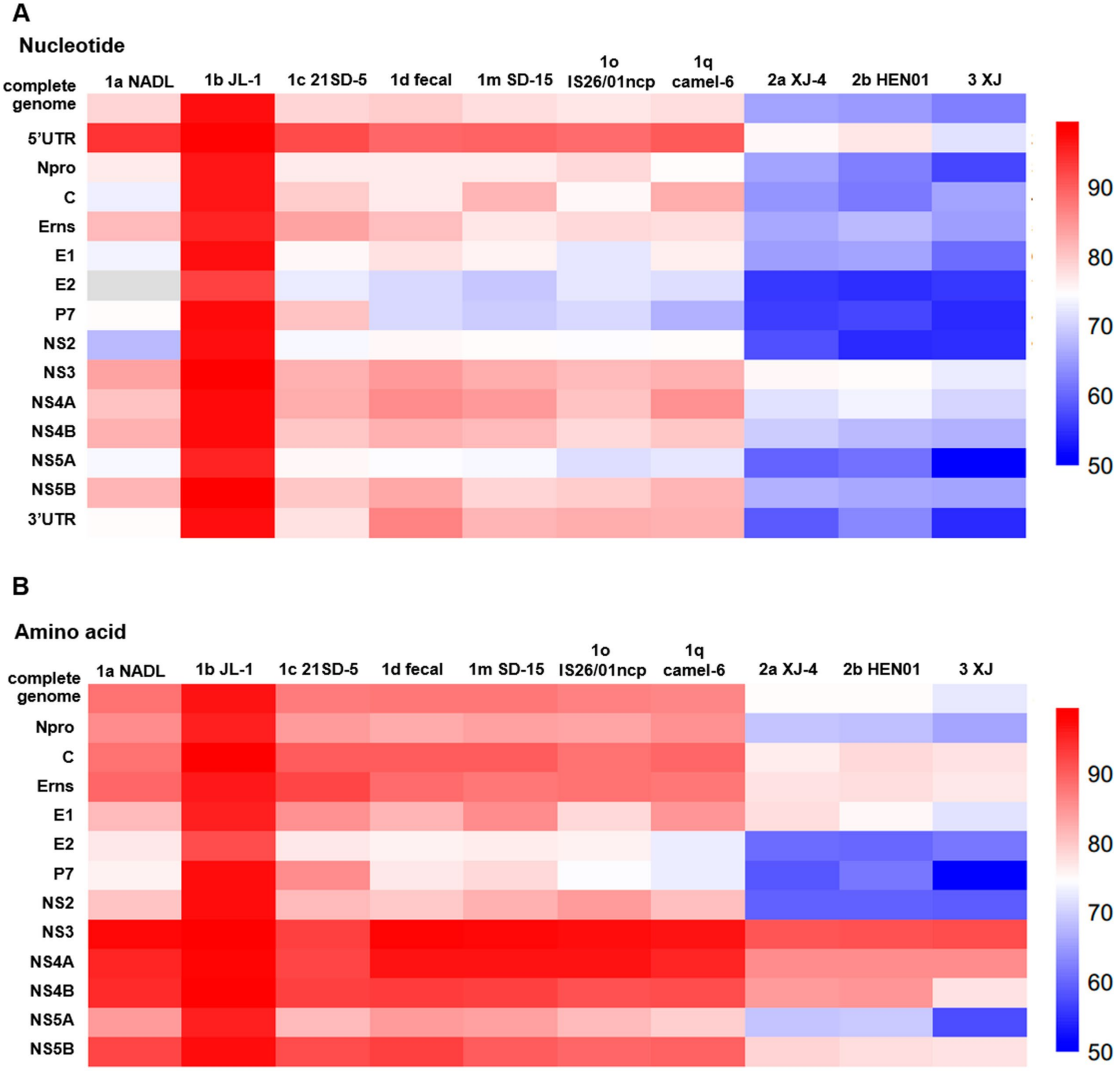
Comparison of nucleotide and amino acid sequence identity with other strains for each fragment. **(A)** Nucleotide sequence identity with other strains. **(B)** Amino acid sequence identity with other strains.

To ascertain the nucleotide identity with other genotype strains, the complete nucleotide sequence was compared with that of 10 representative BVDV isolates, as detailed in Supplementary Table S4. The results demonstrated that BJ-11 exhibited 92.9% homology with BVDV 1b JL-1, the most closely related strain. The homology with other representative BVDV subtype isolates ranged from 68 to 80.4%. The 5′UTR was found to be the most conserved region of the genomes, with nucleotide homology ranging from 74.9 to 93.9% (Figure 5A). Amino acid homology analyses demonstrated that the NS3 was as high as 90.9–99.4% for the three subtypes of BVDV. The homologies of the other genes are shown in Supplementary Table S4 and Figure 5B. Based on these comparisons, the isolate BJ-11 shares the closest genetic relationship with BVDV 1b JL-1, consistent with. The results of phylogenetic analyses.

### 3.8 Sequence analyses of 5′UTR

**Figure 6:**
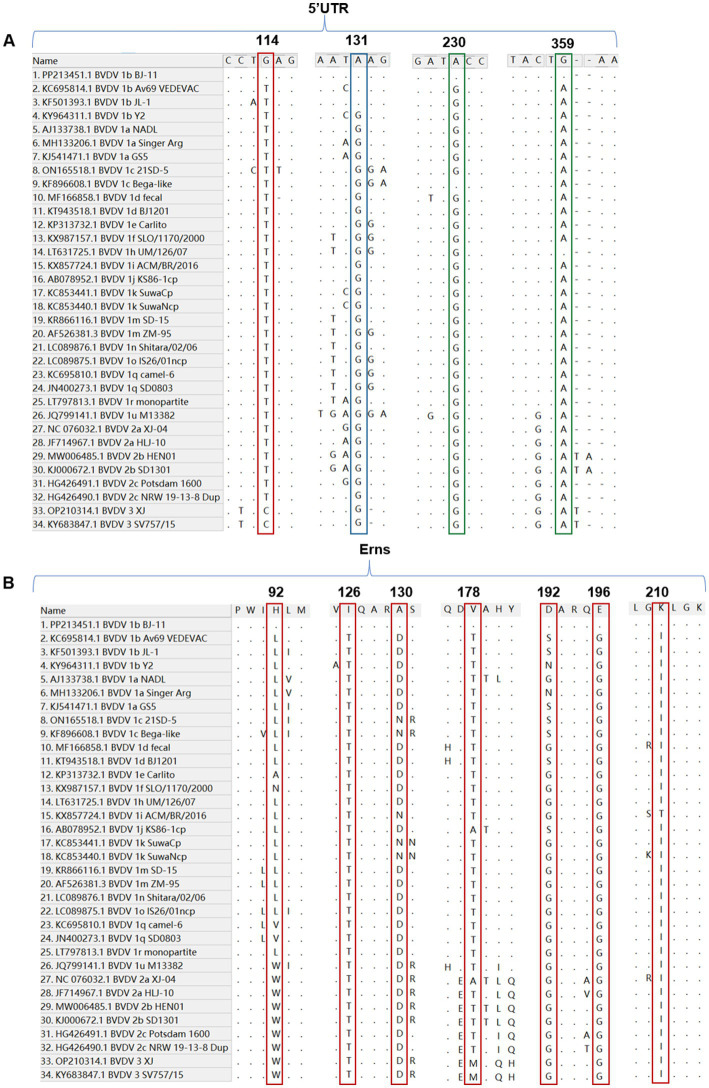
Nucleotide and amino acid sequence with other 33 reference isolates. **(A)** Sequence alignment of the 5’UTR of BVDV BJ-11 and 33 reference strains. The special mutation positions are indicated by boxes. **(B)** Amino acid sequence alignments of Erns and 33 reference isolates. The special mutation positions are indicated by boxes.

For the 5′UTR, the BJ-11 isolate displayed a unique nucleotide mutation at the position 114, where BJ-11 had a G (G^114^), while all other strains exhibited either a T or C (T^114^ or C^114^) (Figure 6A). In addition, a distinguishing feature of the BVDV 1b subtype was identified at position 113. Unlike other subtypes, which consistently had a G (G^113^), BVDV 1b strains showed an A (A^113^), except for KY964311.1 BVDV 1b Y2 which retained G^113^. Further differences were observed in two amino acid positions with the genome. At position 230, Both1b BJ-11 and 1c Bega-like strains displayed an A, whereas the other strains exhibited a G. Similarly, at position 359, the 1b BJ-11 and 1 h UM/126/07 strains displayed a G, whereas the other strains exhibited an A (Figure 6A).

### 3.9 Amino acid analyses of Erns

A comparative analysis of the amino acid sequences of isolate BJ-11 and other reference strains of E0 revealed several unique features. Notably, amino acids at positions 126 and 196 were identified as T and G in other strains, but in BJ-11 these positions were substituted with I and E, respectively. Additionally, discrepancies were observed at the position H92, A130, V178, D192, and K210, where BJ-11 differed from those of reference strains (Figure 6B).

### 3.10 Amino acid analyses and epitope prediction of E2

**Figure 7:**
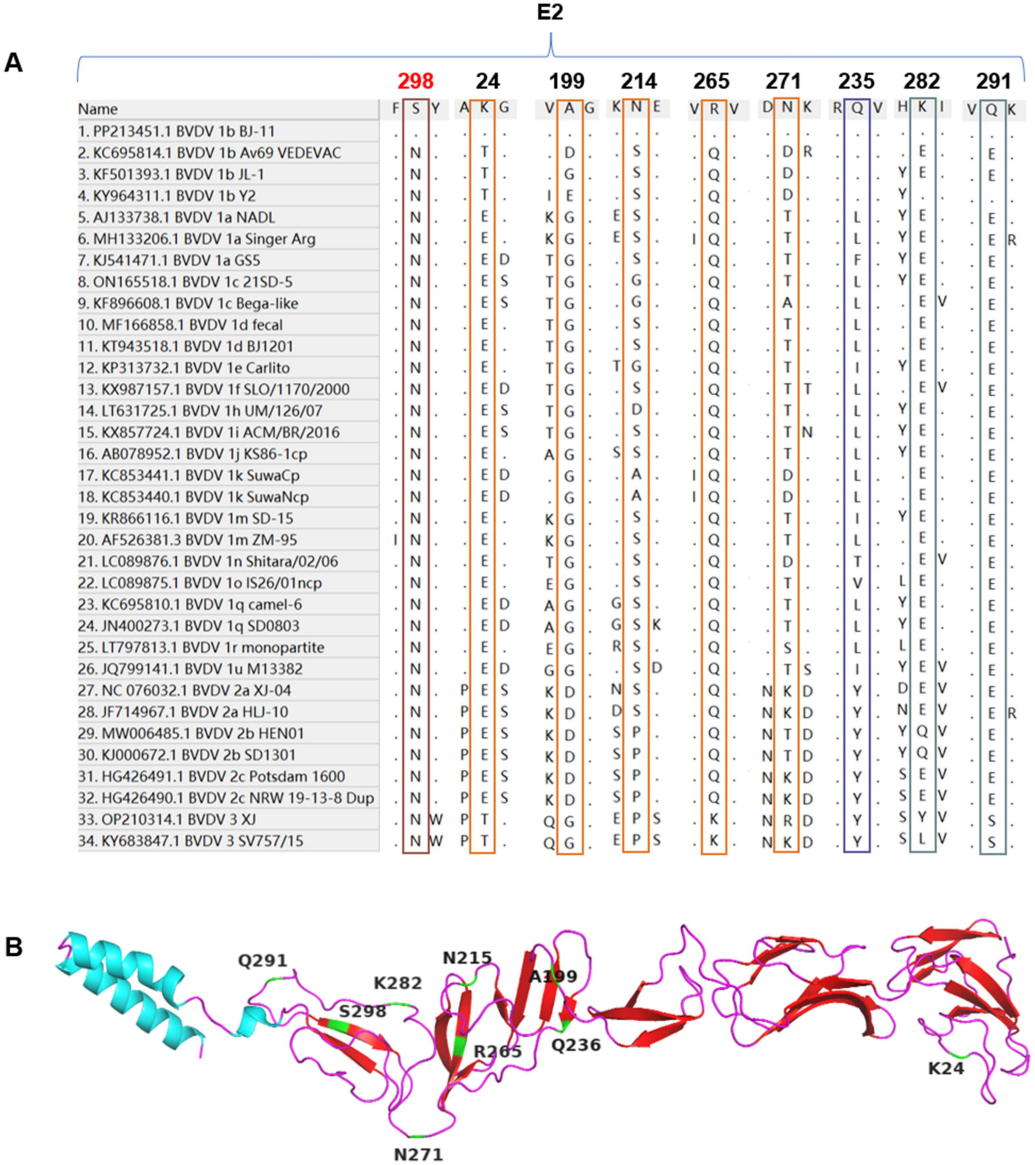
Alignment of E2 amino acid sequences. **(A)** Amino acid sequence alignments of E2 and 33 reference isolates. The special mutation positions are indicated by boxes. **(B)** The cartoon schemes of the BVDV BJ-11 E2 protein structure. The mutations are marked individually.

Alignment analyses of the E2 amino acid sequences from BJ-11 and other representative strains revealed unique molecular characteristics. Differences were observed at position K24, A199, N214, R265, N271, and N298, where BJ-11 differed from other strains. Additionally, residues Q235, K282, and Q291 were similar to those found in multiple BVDV-1b strains but distinct from other strains (Figures 7A,B). Potential linear B-cell epitopes on the E2 protein were predicted using BepiPred-2.0.^6^

**Figure 8:**
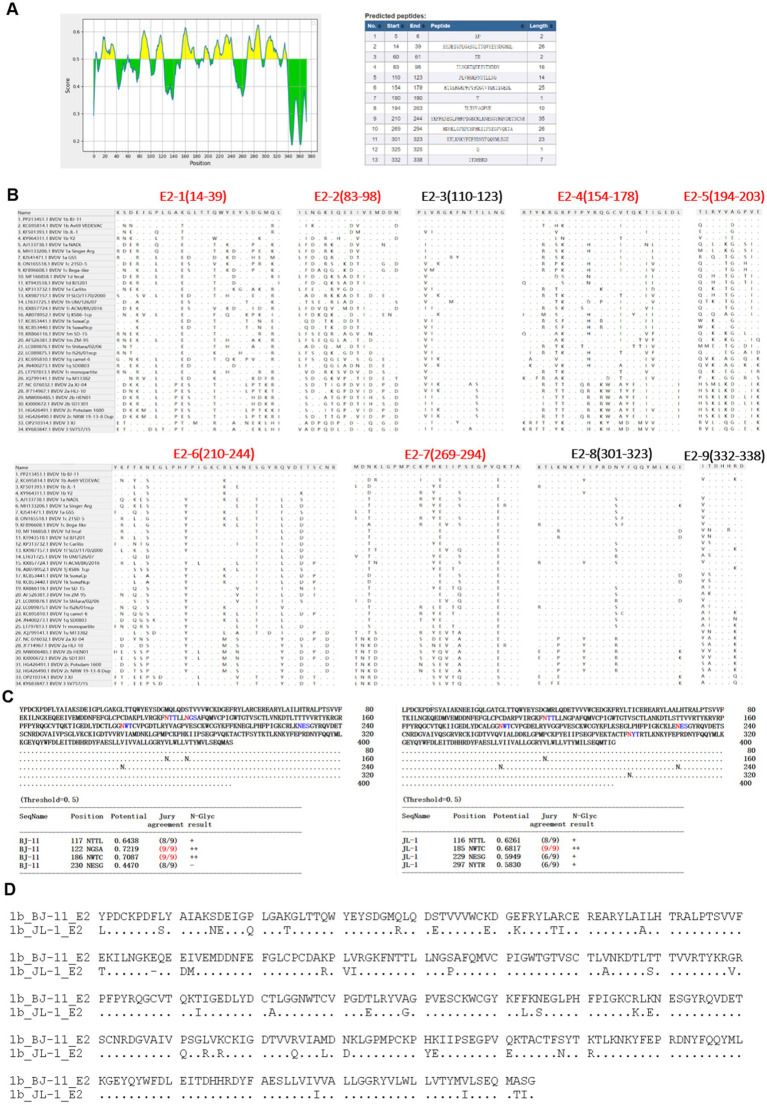
Amino acid characteristics of E2 protein. **(A)** Epitope prediction of E2. **(B)** Display of E2 epitope, multi-variable zones are in red. **(C)** Prediction of glycosylation sites for E2 of BJ-11 and JL-1. **(D)** The alignment of BJ-11 and JL-1 E2 gene.

As illustrated in Figure 8A, nine potential linear B cell epitopes were identified, with one region showing the greatest variation. Only the E2-3, E2-8, and E2-9 regions were conserved (Figure 8B). The E2 glycoprotein, known to be the most immunodominant glycoprotein in BVDV, displayed unique glycosylation site in BJ-11. Prediction using NetNGlyc 1.0 (see text footnote 3), had three glycosylation sites located at positions 117NTT, 122NGS, and 186NWT. One of the glycosylation sites, however, exhibited a mutation T232S, which reduced glycosylation activity at the NTS site, whereas the JL-1 E2 glycosylation prediction included 116NTT, 185NWT, 229NES, and 297NYT (Figure 8C). A comparative analysis of the amino acid sequences of BJ-11 and JL-1 E2 revealed 46 mutations in addition to a single amino acid insertion (Figure 8D).

### 3.11 Recombination analyses

**Figure 9:**
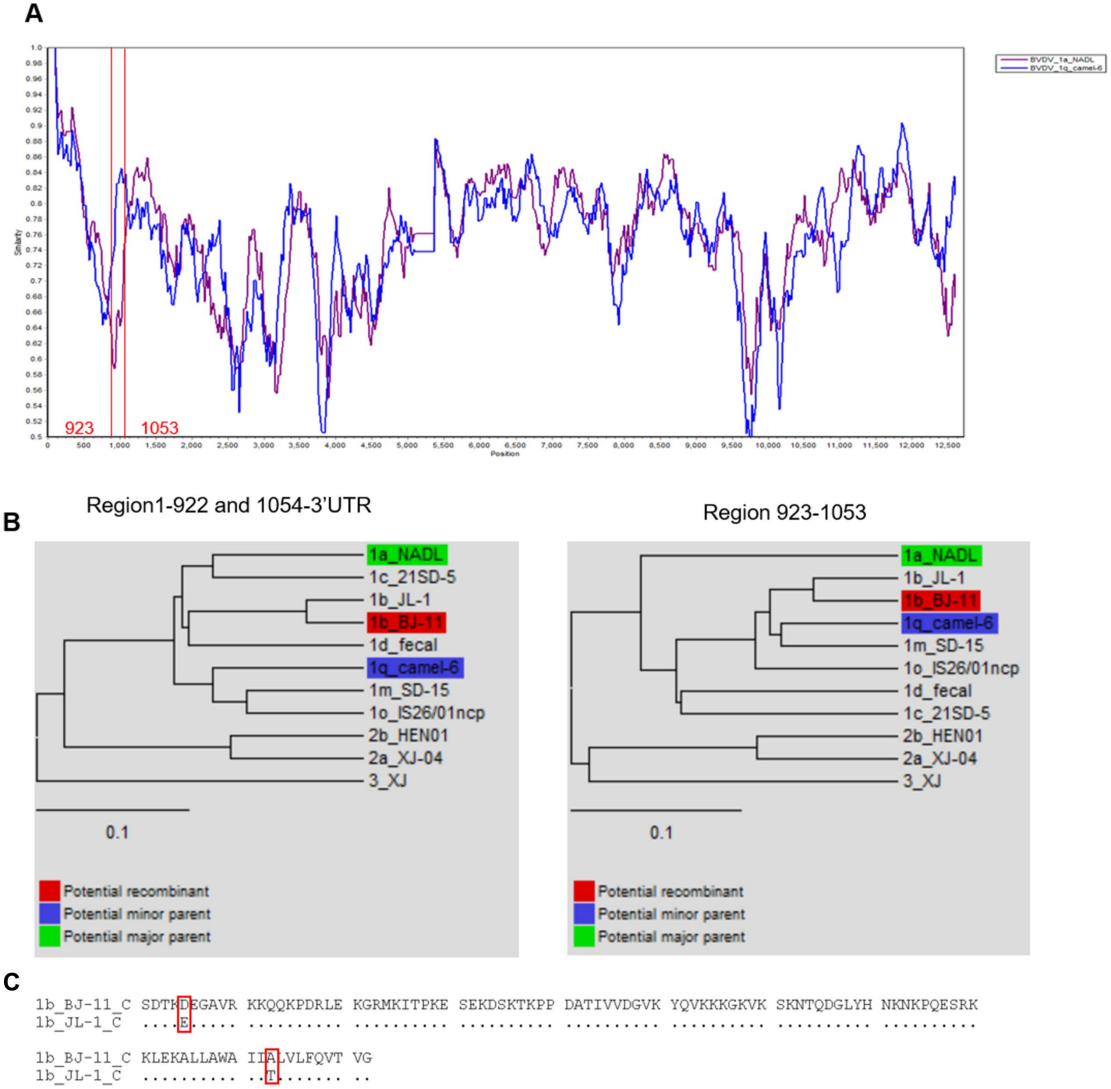
Recombination analysis of BVDV BJ-11 strain genome. **(A)** Similarity plots and boot scanning analyses were performed using the SimPlot recombination breakpoint is shown with red lines. **(B)** Phylogenies show recombination event of the major and minor parent regions. **(C)** Comparison of BJ-11 recombinant region C protein of BJ-11 and JL-1.

To investigate genetic recombination of BJ-11, the complete genome was aligned against 10 representative BVDV genomes (Supplementary Table S5) using the ClustalW tool in MEGA 11. Potential recombination events were identified using the RDP4 program. A single recombination event was detected, and its parental strains were identified as BVDV-1a NADL and BVDV 1q Camel-6 (Table 1). Similarity analysis supported the potential signatures of genetic exchange at nucleotides 922–1,054 (Figures 9A,B). Additionally, the amino acid sequence comparison in the recombinant region of C revealed the presence of two amino acid mutation sites when comparing BJ-11 and JL-1 (Figure 9C).

**Table 1:** Subtypes, titer, Kd and Kaff of monoclonal antibodies.

Recombinant	Location(s) [nt (99%CI)]	Major parent	Minor parent	Methods						
				RDP	GeneConv	Bootscan	MaxChi	Chimaera	SiScan	3Seq
	920–1,048	NADL 1a	Camel-6 1q	5.354 × 10^−03^	-	3.669 × 10^−03^	-	-	1.089 × 10^−02^	-

## 4 Discussion

Since it was first described in 1946, BVDV has spread worldwide, causing significant economic losses to the livestock industry (Olafson et al., 1946). In light of their significant economies, several countries in Europe and the Americas are currently engaged in mandatory or voluntary BVD control and/or eradication programs. Vaccination is the primary measure of protection for cattle against the disease, with most commercially available vaccine targeting BVDV 1a and BVDV 2a. Research has shown that the BVDV1a vaccine provides protection against BVDV1b (Xue et al., 2011; Leyh et al., 2011; Givens et al., 2012). However, other studies have indicated that the level of protection may be inadequate owing to discrepancies between vaccine and circulation field strains (Walz et al., 2018; Mosena et al., 2020). For example, viral RNA was detected in fetal tissues 30 days after expose to HoBi-like viruses, despite high neutralizing titers of 1,448–5,793 (Bauermann et al., 2017). Similarly, the cow from which the BJ-11 strain was isolated had also been vaccinated with an inactivated vaccine, yet remained infected with BVDV and ultimately succumbed to severe diarrhea and a multitude of complications.

Calves infected with BJ-11 exhibited a range of respiratory and digestive symptoms. The immunosuppression caused by acute BVDV infection is characterized by reduced Lym levels and impaired Leu function (Liu et al., 2018; Palomares et al., 2014). Neus, the most abundant of disease, contributing to secondary bacterial infections and increased morbidity and mortality (Abdelsalam et al., 2023; Ma et al., 2021). Severe lung lesions observed in BJ-11-infected calves further support the classification of BVDV as part of the bovine respiratory disease complex (BRDC), a significant health challenge in young cattle (Zhou et al., 2023). Lesion in the spleen and tonsils, critical immune organs, indicate immune dysfunction, which is consistent with previous reports (Oguejiofor et al., 2019). The JL-1, which is the closest to the BJ-11 strain based on the evolutionary analysis, was first reported in 2014. Experimental studies showed that JL-1 primarily caused depression, fever, leukopenia, and viremia, along with significant morphological alterations in the gastrointestinal tract and lymphoid tissues (Zhang et al., 2014). Notably, JL-1 infection lacked diarrhea or pulmonary symptoms, which are typically regarded as relatively mild symptoms of BVDV infection. In contrast, the BJ-11 strain has evolved into a highly pathogenic variant, causing severe diarrhea, bloody stools, and even mortality in addition to severe pulmonary symptoms. This stark difference highlights the genetic evolution of BJ-11, transforming it into a potent and dangerous strain with increased virulence.

In challenge experiments, BJ-11 has been shown to induce acute lung injury, characterized by pulmonary hemorrhage and extensive inflammatory cell infiltration. This is likely a significant contributing factor to mortality. Research indicates that acute lung injury caused by PRRSV (porcine reproductive and respiratory syndrome virus) is primarily attributed to viral virulence and vaccine strain characteristics (Sun et al., 2024). The inactivated BVDV vaccines currently in use do not provide adequate protection against wild circulated strains. This lack of efficacy drives viral mutations, enabling adaptation to the host and evasion of the immune response. Ineffective vaccination can also fail to clear the virus, prolonging infection and facilitating viral transmission. Furthermore, the low level of protection offered by these vaccines may unintentionally create selective pressure that favors the survival and proliferation of more virulent strains (Read et al., 2015). Although vaccines are effective for eradicating the virus, they do not guarantee successful protection against BVDV. Therefore, vaccines should be used rationally in conjunction with biosecurity, serological and genetic monitoring, control of secondary bacterial infections, and production management. The aim of this approach is twofold: to alleviate clinical signs in cattle and to provide effective protection against the spread of the pathogen.

Multigene evolutionary analyses provide a reliable approach for viral classification. In this study, BJ-11 isolate was analyzed using sequences from multiple genomic regions, including the 5′UTR, complete genome sequence, Npro, NS3, E0, and E2. Among these, the 5′UTR was identified as the most conserved region of the genome in the sequence comparisons, which may explain its widespread use as a target for PCR amplification for the identification of BVDV. Additionally, the nonstructural protein 3 (NS3), NS4A, and NS4B genes exhibited greater conservation compared to other regions of the BVDV genome.

Genetic recombination is critical role in viral evolution, serving as a primary source of genetic variation and the evolution of viral genomes (Simon-Loriere and Holmes, 2011; Guo et al., 2021). In the absence of selective pressure, recombination occurs randomly; however, only variants with high adaptive capacity are likely to persis. This process not only increases the risk of enhanced virulence. Notably, homologous recombination has been reported more frequently in BVDV than in other members of the *Flaviviridae* family (Shi et al., 2012). In this study, a recombination signal was observed with the parents NADL 1a (GenBank Accession Number: AJ133738.1) and Camle-6 (Gene Registry Number: KC695810.1). NADL 1a was initially isolated in the United States and has become a commonly used vaccine strain and standard strain for research purposes. Camel-6 was isolated from camels in Lanzhou, China in 2014. A study on the origin and dissemination of BVDV-1 indicated that the virus might have been introduced in China during the 1960s. Although animal husbandry advanced after the establishment of the People’s Republic of China, the livestock industry experienced rapid development only after economic reforms in the early 1980s and China’s accession to the WTO in 2001. China imports a considerable number of live animals. Cows of superior breeding imported from abroad were initially transported to Beijing and subsequently to Qinghai. Regular contact between animals from different regions resulted in the dissemination and exchange of viruses.

BVDV exhibits a high mutation rate owing to the absence of RNA polymerase proofreading activity during viral genome replication. The E0, a BVDV membrane protein with high antigenic conservation and neutralizing of epitopes, plays a pivotal role in preventing host infection by the virus. This is achieved mainly by blocking the synthesis of interferon (IFN), which in turn hinders the innate immune defenses of the host, thus facilitating long-term infection. It can be employed as a genetically engineered diagnostic antigen. Residues W33, L71, Q127, N130, S145, G148, T102, and D107 in CSFV are essential for antibody binding (Lin et al., 2010). In this study, a mutation at residue N130 (N130A) was identified in BJ-11, which may have impacted vaccine efficacy. The E2 protein, a key receptor-binding component of BVDV, contains the majority of the antigenic determinants of the virus and elicits the production of neutralizing antibodies (Bhuyan et al., 2018). However, the E2 gene is highly unstable and prone to mutations, exhibiting the highest mutation rate among all regions of BVDV. Prediction of epitopes indicated that five out of the nine epitopes were located in the hypervariable region, which ultimately resulted in a reduction in the efficacy of the vaccine.

Protein glycosylation, a prevalent yet intricate post-translational modification, exerts considerable influence on protein functionality, stability, subcellular localization, and other characteristics (Eichler, 2019). The glycosylation sites of viral membrane proteins exert a significant influence on the pathogenicity and antigenicity of the viruses. The E2 glycoprotein is the most immunodominant viral protein in *Pestiviruses* and has the capacity to impede viral infections. Four glycosylation sites have been identified: N117, N186, N230, and N298 (Mirosław and Polak, 2020). Pande A reported that the deletion of the E2-298 glycan site subsequently diminishes the capacity of the recombinant E2 protein to impede viral infection, implying that this reduction increases the likelihood of viral infection (Pande et al., 2005). In our analysis of BJ-11 strain, the N298 glycosylation site was found to be absent, which may have enhanced the infectivity. Additionally, the glycosylation sites, N124 and P124S, were added to the NGS site. Furthermore, the NET at position 230 was replaced by an NES. Replacing serine with threonine at this site markedly reduces the efficiency of N-glycosylation. Moreover, this mutation is specific to BVDV 1b and BVDV 2 (Malaby and Kobertz, 2013).

In conclusion, we successfully isolated a BJ-11 strain from the feces of calves that died from severe diarrhea. Virulence experiments demonstrated that this strain exhibited acute clinical symptoms and even death. Genomic comparisons and phylogenetic analysis revealed that the virus was BVDV 1b, which is the predominant genotype in China. The closest related strain, JL-1, was first isolated in 2014, and BJ-11 may present an evolved, more virulent form of JL-1. The increased virulence of BJ-11 was analyzed through comparison with JL-1 strain using pathogenicity data and gene analyses. These findings provide important insights into the genetic evolution of BVDV and offer a foundation for improving vaccine development and prevention strategies. The results also highlight a critical question: is viral evolution and increased virulence driven by imperfect vaccines, or other factors? Future efforts should focus on the development of novel vaccines with broad protection, improved immunization strategies, and enhanced biosecurity measures. Furthermore, continuous monitoring of viral mutations and a deeper understanding the epidemiology of BVDV will be essential for the control and prevention of the emergence of high pathogenicity strains.

## Data Availability

The complete genomic sequence of the BVDV strain was assembled using the next-generation sequencing (NGS) method (28). The BVDV genome sequence was submitted to GenBank (accession number PP213451.1).

## Footnotes

^1^ https://www.lynnon.com/dnaman.html

^2^ https://sray.med.som.jhmi.edu/SCRoftware/SimPlot/#downloads

^3^ https://services.healthtech.dtu.dk/services/NetNGlyc-1.0/

^4^ https://swissmodel.expasy.org/interactive

^5^ http://www.pymol.org/

^6^ https://services.healthtech.dtu.dk/services/BepiPred-2.0/
