# Supplementary material for: Highly pathogenic bovine viral diarrhea virus BJ-11 unveils genetic evolution related to virulence in calves
Source: Front Microbiol. 2025 Jan 14;15:1540358. doi: 10.3389/fmicb.2024.1540358 (PMC11772275; doi:10.3389/fmicb.2024.1540358)
Supplement: Supplementary file 1 [file Data_Sheet_1.docx]

Supplementary Material

# Supplementary Figures and Tables

## Supplementary Figures


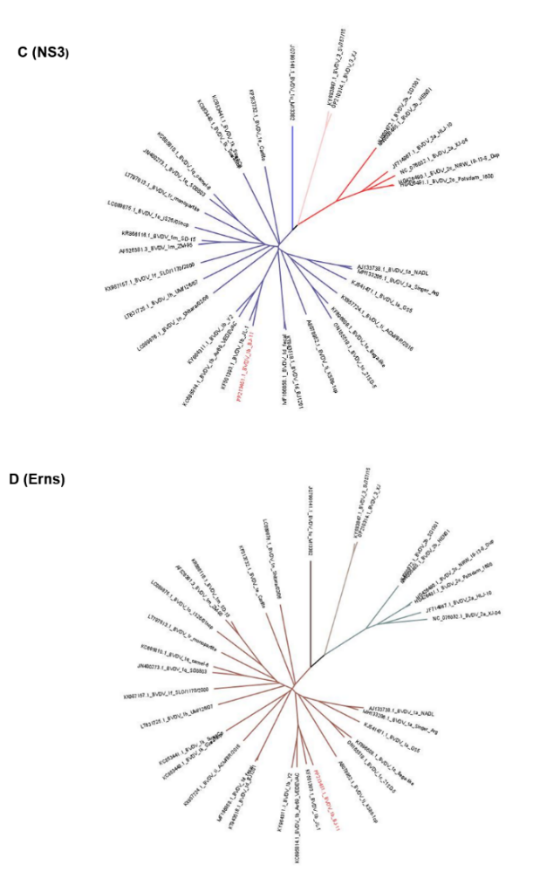

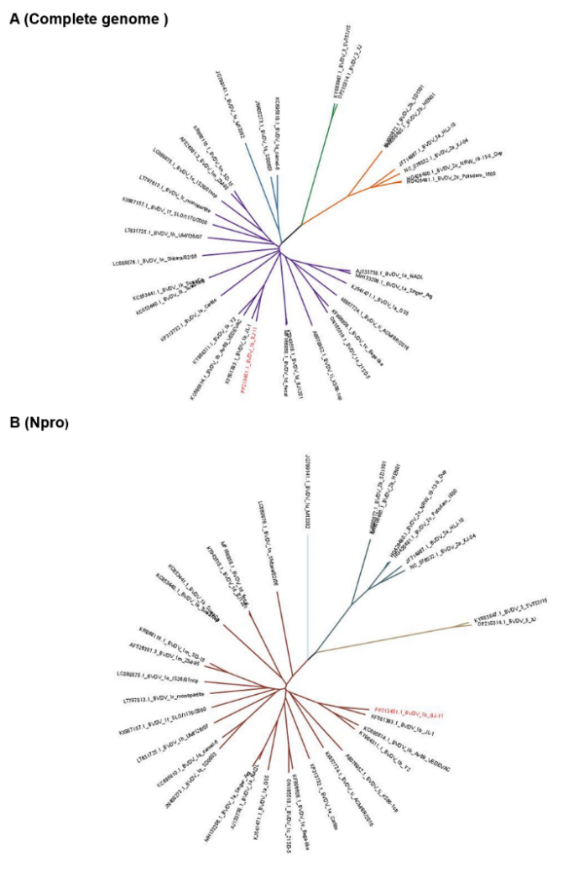


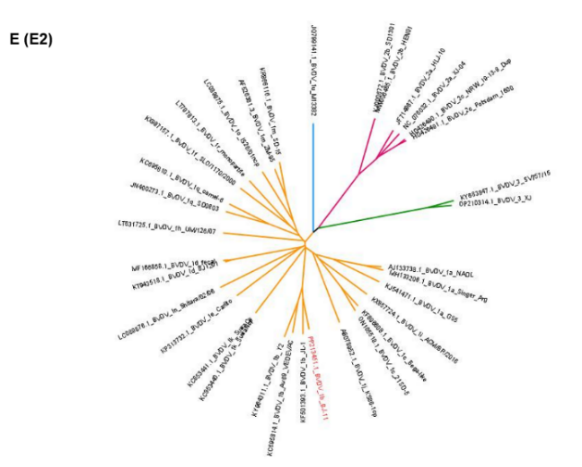


**Supplementary Figure 1.** Phylogenetic analysis of BVDV BJ-11. **(A-E)** Phylogenetic analysis of complete gene, Npro, NS3, Erns, and E2. BJ-11 strain isolated in this study (highlighted in red).

**1.2. Supplementary tables**

**Table S1.** Primers used for amplification of the complement genome sequence.

|  | Primers | | Primer sequence (5’-3’) | Length (nt) |
| --- | --- | --- | --- | --- |
| BVDV | | F: | CATACCTTCAGTAGGACGAGC | 280 |
|  | | R | ATGTGCCATGTACAGCAGAG |  |
| BoHV-1 | | F: | TGAGGCCTATGTATGGGCAGTT | 432 |
|  | | R | GGACACAACAAACAATGCGG |  |
| BRoV A/C | | F: | GCATGGATGAAATGGTTAGAGA | 467 |
|  | | R | TGGATTGAAGTACCATGTAGT |  |
| BRoV B | | F: | ACAGTGAATGCTTGCGTCAG | 584 |
|  | | R | GCTTCCATGCCTGAAACACA |  |
| BCoV | | F: | GCCGATCAGTCCGACCAATC | 407 |
|  | | R | AGAATGTCAGCCGGGGTAT |  |
| BPIV-3 | | F: | TGTCTTCCACTMGATAGAGGGATAAAATT | 203 |
|  | | R | CCTTTTTCATCTAGAATCTGAACTACTCC |  |
| BRSV | | F: | ACACATCAATYCAAAGCACCACAC | 374 |
|  | | R | GCTRGTTCTGTGGTGGRTTGTTGTC |  |
| M. bovis | | F: | CCTTTTAGATTGGGATAGCGGATG | 360 |
|  | | R | CCGTCAAGGTAGCATCATTTCCTAT |  |
| BVDV | | RT-qPCR-F | CCGCGAMGGCCGAAAAGA |  |
|  | | RT-qPCR-R | TGACGACTNCCCTGTACTCAG |  |

**Table S2.** Clinical signs and designated score.

|  | Score | Clinical signs |
| --- | --- | --- |
| Conjunctivitis | 0 | None (Normal, light pink conjunctiva, no congestion) |
|  | 1 | Slight (Conjunctiva bright red or dark red) |
|  | 2 | Moderate (Conjunctiva is flushed, inflamed, watery, swollen, and has discharge) |
|  | 3 | Severe (Conjunctiva is red, swollen, congested, and has a syrupy, mucous, or mucopurulent discharge) |
| Nasal discharge | 0 | None (No discharge) |
|  | 1 | Slight (Dry nose, runny nose or blisters on nose) |
|  | 2 | Moderate (Thick, greyish-white, opaque nasal discharge) |
|  | 3 | Severe (Mucous, purulent, yellowish-white nasal discharge with a foul odor) |
| Coughing | 0 | None (No cough) |
|  | 1 | Slight (Occasional cough) |
|  | 2 | Moderate (Frequent cough) |
|  | 3 | Severe (Frequent cough, short, weak cough, neck extension and head shaking) |
| Diarrhea | 0 | None (Somewhat shaped, dark green, straw hat suit stacking) |
|  | 1 | Slight (Yellowish-white, porridge-like and foul-smelling feces) |
|  | 2 | Moderate (Watery feces, thin pasty feces, watery feces containing blood, mucus, or diarrheal feces containing bright red blood, blood clots) |
|  | 3 | Severe (thin soupy or watery feces with curds, blood, blood clots and gas bubbles, fishy smell) |
| Appetite | 0 | Normal (Desire to eat at normal mealtimes) |
|  | 1 | Decreased (Slight loss of appetite, fussy eating) |
|  | 2 | Strongly reduced (Loss of appetite, loss of appetite) |
|  | 3 | Anorexia (Marked decrease in appetite, even complete loss of appetite) |

**Table S3.** Complete genomic information.

| Region | Nucleotide (nt) | Length (nt) | Protein | Length (aa) |
| --- | --- | --- | --- | --- |
| 5'UTR | 1-382 | 382 |  |  |
| Npro | 383-886 | 504 | p20 | 168 |
| Capsid | 887-1192 | 306 | p14 | 102 |
| Erns | 1193-1873 | 681 | gp48 | 227 |
| E1 | 1874-2458 | 585 | gp25 | 195 |
| E2 | 2459-3580 | 1122 | gp53 | 374 |
| P7 | 3581-3790 | 210 | p7 | 70 |
| NS2 | 3791-5149 | 1359 | p54 | 453 |
| NS3 | 5150-7198 | 2049 | p80 | 683 |
| NS4A | 7199-7390 | 192 | p10 | 64 |
| NS4B | 7391-8431 | 1041 | p32 | 347 |
| NS5A | 8432-9919 | 1488 | p58 | 496 |
| NS5B | 9920-12,079 | 2160 | p75 | 720 |
| 3'UTR | 12,080-12,246 | 167 |  |  |

**Table S4.** A comparative analysis of the isolate genotypes with other representative strains (%).

|  | 1a NADL | 1b  JL-1 | 1c  21SD-5 | 1d  fecal | 1m  SD-15 | 1o IS26/01ncp | 1q Camel-6 | 2a  XJ-4 | 2b HEN01 | 3  XJ |
| --- | --- | --- | --- | --- | --- | --- | --- | --- | --- | --- |
| Nucleotide |  |  |  |  |  |  |  |  |  |  |
| complete genome | 79.8 | 92.9 | 79.8 | 80.4 | 79.2 | 78.7 | 79.3 | 70.6 | 70 | 68 |
| 5'-UTR | 90.8 | 93.9 | 89.3 | 87.4 | 87.6 | 87.1 | 88.1 | 77.5 | 78.7 | 74.9 |
| Npro | 78.4 | 92.5 | 78.2 | 78.2 | 78.4 | 79.6 | 77 | 70.6 | 67.9 | 64.3 |
| C | 75.8 | 92.5 | 80.4 | 78.2 | 82 | 77.4 | 82.7 | 69.6 | 67.6 | 70.6 |
| Erns | 81.6 | 91.5 | 83.4 | 81.5 | 78.6 | 79.6 | 79.3 | 70.8 | 72.2 | 70.2 |
| E1 | 76.1 | 93 | 77.3 | 78.8 | 77.8 | 75.2 | 77.9 | 70.3 | 70.6 | 66.8 |
| E2 | 74,8 | 89.8 | 75.6 | 74.4 | 73.1 | 75.1 | 74.7 | 63.4 | 62.9 | 63.5 |
| P7 | 77.1 | 93.3 | 81 | 74.3 | 73.3 | 74.3 | 71.4 | 63.8 | 64.3 | 62.4 |
| NS2 | 72.2 | 92.8 | 76.3 | 77.3 | 77.1 | 76.7 | 77.1 | 64.8 | 62.5 | 62.7 |
| NS3 | 83.2 | 94.3 | 82.4 | 84 | 82.7 | 81.6 | 82.2 | 77.4 | 77.2 | 75.4 |
| NS4A | 81.2 | 93.2 | 82.8 | 84.9 | 83.9 | 81.2 | 84.4 | 75 | 76 | 74 |
| NS4B | 82.4 | 93.2 | 80.7 | 82.3 | 81.7 | 79.5 | 80.8 | 73.2 | 72.2 | 71.4 |
| NS5A | 76.5 | 91.5 | 77.3 | 76.9 | 76.4 | 74.7 | 75.3 | 66.3 | 67 | 59.2 |
| NS5B | 81.9 | 94.6 | 80.9 | 82.9 | 79.9 | 80.4 | 81.9 | 71.5 | 70.8 | 70.4 |
| 3'-UTR | 77.2 | 92.8 | 79 | 85.5 | 82 | 82.6 | 82.4 | 65.5 | 68.7 | 62.6 |
| Amino acid |  |  |  |  |  |  |  |  |  |  |
| complete genome | 88.1 | 96.9 | 87.2 | 88 | 87.9 | 87 | 86.4 | 75.1 | 75.1 | 72.4 |
| Npro | 85.7 | 95.8 | 84.5 | 82.7 | 83.9 | 83.3 | 85.1 | 69 | 68.5 | 66.1 |
| C | 88.2 | 99.3 | 90.2 | 90.1 | 90.2 | 88.1 | 89.2 | 76.5 | 78.4 | 77.2 |
| Erns | 89.4 | 96.2 | 92.1 | 89 | 87.7 | 88.1 | 87.7 | 77.5 | 78 | 77.1 |
| E1 | 81.5 | 95.6 | 85.1 | 82.1 | 85.6 | 78.5 | 84.6 | 77.9 | 75.4 | 71.8 |
| E2 | 76.9 | 91.8 | 76.7 | 76.1 | 76.4 | 76 | 73.2 | 60.4 | 60.1 | 61.6 |
| P7 | 75.7 | 97.1 | 85.7 | 77.1 | 78.6 | 74.3 | 72.9 | 58.6 | 61.4 | 50 |
| NS2 | 80.6 | 97.1 | 81.5 | 80.1 | 82.3 | 84.1 | 80.8 | 59.4 | 59.8 | 59.2 |
| NS3 | 97.8 | 99.4 | 92.5 | 98 | 97.8 | 97.1 | 96.9 | 90.9 | 91.1 | 91.9 |
| NS4A | 95.3 | 98.4 | 92.2 | 96.9 | 96.9 | 96.9 | 95.3 | 85.9 | 85.9 | 85.9 |
| NS4B | 94.5 | 98.5 | 92.5 | 93.4 | 92.8 | 91.1 | 91.6 | 84.1 | 85 | 77.2 |
| NS5A | 84.1 | 95.8 | 81.5 | 84.1 | 83.7 | 81.4 | 79.2 | 68.8 | 69.6 | 57.7 |

**Table S5.** Reference viruses used for homology and recombination analysis.

| Subtype | Strain | GenBank accession number |
| --- | --- | --- |
| BVDV 1a | NADL | AJ133738.1 |
| BVDV 1b | JL-1 | KF501393.1 |
| BVDV 1c | 21SD-5 | ON165518.1 |
| BVDV 1d | fecal | MF166858.1 |
| BVDV 1m | SD-15 | KR866116.1 |
| BVDV 1o | IS26/01ncp | LC089875.1 |
| BVDV 1q | camel-6 | KC695810.1 |
| BVDV 2a | XJ-04 | NC_076032.1 |
| BVDV 2b | HEN01 | MW006485.1 |
| BVDV 3 | XJ | OP210314.1 |
